# Epidemiology of the human immunodeficiency virus in Saudi Arabia; 18-year surveillance results and prevention from an Islamic perspective

**DOI:** 10.1186/1471-2334-4-25

**Published:** 2004-08-06

**Authors:** Tariq A Madani, Yagob Y Al-Mazrou, Mohammad H Al-Jeffri, Naser S Al Huzaim

**Affiliations:** 1 Department of Medicine, King Abdulaziz University, Jeddah; 2 Ministry of Health, Riyadh, Saudi Arabia

## Abstract

**Background:**

data on HIV epidemiology and preventive measures in Islamic countries is limited. This study describes the results of 18-year of HIV surveillance in Saudi Arabia (SA) and the preventive measures implemented from an Islamic perspective.

**Methods:**

surveillance for HIV has been underway in SA since 1984. Indications for HIV testing include clinical suspicion, screening of contacts of HIV-infected patients, and routine screening of blood and organ donors, prisoners, intravenous drug users, patients with other sexually transmitted infections, and expatriates pre-employment. This is a case series descriptive study of all confirmed HIV infections diagnosed in SA from 1984 through 2001.

**Results:**

a total of 6046 HIV infections were diagnosed, of which 1285 (21.3%) cases were Saudi citizens. Over the 18-year surveillance period the number of HIV infections diagnosed annually among Saudi citizens gradually increased and, over the period 1997–2001, it reached to 84 to 142 cases per year. The number of cases per 100,000 population varied widely between regions with a maximum of 74 cases and a minimum of 2 cases. The infection was most common in the age group 20–40 years (74.6%) and predominantly affected men (71.6%). The modes of transmission among Saudi citizens and expatriates, respectively, were as follows: heterosexual contact, 487 (37.9%) and 1352 (28.4%) cases; blood transfusion, 322 (25.0%) and 186 (3.9%) cases; perinatal transmission, 83 (6.5%) and 19 (0.4%) cases; homosexual contact, 32 (2.5%) and 38 (0.8%) cases; intravenous drug use, 17 (1.3%) and 33 (0.7%) cases; bisexual contact, 10 (0.8%) and 14 (0.3%) cases; unknown, 334 (26.0%) and 3119 (65.5%) cases. The number of HIV infections transmitted by blood or blood products transfusion declined to zero by year 2001 and all such infections occurred due to transfusions administered before 1986. At HIV diagnosis, 4502/6046 (74.5%) patients had no symptoms, 787 (13.0%) patients had non-AIDS defining manifestations, and 757 (12.5%) patients had AIDS. A total of 514/1285 (40%) Saudi patients died by year 2001.

**Conclusions:**

the number of HIV cases in SA is limited with heterosexual contact being the main mode of transmission. From an Islamic perspective, preventive strategies include prevention of non-marital sex and intravenous drug use with encouragement of "safe sex" through legal marriage.

## Background

The human immunodeficiency virus (HIV) continues to be a major health problem worldwide despite enormous efforts to control its spread. As of December 2002, the estimated number of people living with HIV is 42 millions [1]. In year 2002 alone, the HIV epidemic claimed more than 3 million lives and an estimated 5 million people acquired HIV [1]. Available data point to increasing HIV infection rates in the Middle East and North Africa, with an estimated 83000 people having acquired the virus, and 37000 having died, in 2002. This brings to 550000 the estimated number of people living with HIV in this region [1]. Sexual contact remains the main mode of transmission of the disease worldwide followed by intravenous drug use and perinatal transmission [1]. Transfusion of blood and blood products is no longer a significant risk factor for acquiring HIV infection since the introduction of routine pre-transfusion HIV screening in 1985–1986 in most countries.

**Figure 1:**
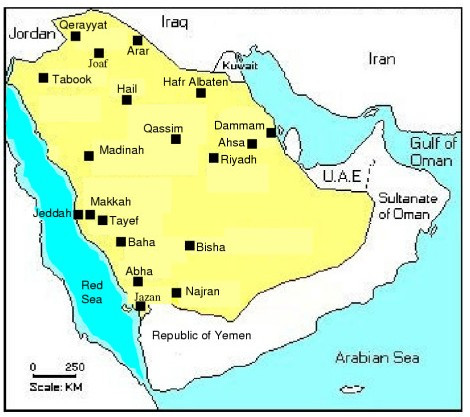
A map of Saudi Arabia showing the main regions and cities where HIV cases were identified.

Saudi Arabia occupies most of the Arabian Peninsula with an area of about 2,240,000 sq km (figure 1). It comprises 13 administrative provinces, namely, Makkah province (which includes the holy city of Makkah, Jeddah and Tayef), Madinah province (which includes the holy city of Madinah), Riyadh province (which includes the capital city Riyadh), the Eastern province (which includes Dammam, Ahsa, and Hafr Albaten), Asir province (which includes Abha and Bisha), Joaf province (which includes Joaf and Qerayyat), Hudud Shamaliyah (North borders) province (which includes Arar), and Baha, Jizan, Najran, Hail, Qassim, and Tabook provinces. The capital and largest city is Riyadh. The population in Saudi Arabia is 24,293,844, including 5,576,076 non-nationals as estimated in July 2003. Approximately, 42.3% of the population is below 15 years of age, 54.8%, between 15 and 64 years, and 2.9%, above 64 years of age. Ninety nine percent of the population is Muslim and the country is governed according to the Islamic law.

Information on HIV epidemiology in Saudi Arabia and other Islamic countries is limited. Islam prohibits non-marital sex, homosexuality, and intravenous drug use. Therefore, the prevalence and incidence of HIV and other sexually transmitted infections are expected to be low in Islamic communities. This study describes the results of HIV surveillance activities that have been underway in Saudi Arabia from 1984 through 2001 and the preventive measures implemented by the government.

## Methods

Surveillance for HIV infections has been underway since 1984 when the first case of HIV was diagnosed in Saudi Arabia. HIV cases are detected by HIV testing for various indications including clinical suspicion, screening of contacts of HIV-infected patients, routine screening of blood and organ donors, and testing of all prisoners, intravenous drug users, patients with other sexually transmitted infections, and expatriates pre-employment. In addition to being HIV tested in their homeland as a compulsory pre-requisite for employment in Saudi Arabia, expatriates are routinely retested for HIV upon arrival to Saudi Arabia before they are allowed to work and then regularly every 2 years to have their legal residence permits renewed. Only HIV-negative expatriates are allowed to work in Saudi Arabia. Routine screening of blood, blood products, and organ donors for HIV has been a standard procedure in Saudi Arabia since 1986. Enzyme-linked immunosorbent assays (ELISA) are used for routine HIV1/HIV2 testing. Positive ELISA results are confirmed by Western blot test performed in selected referral laboratories. The expanded World Health Organization (WHO) case definition was used to define AIDS [2]. All HIV infections diagnosed in governmental or private health care facilities are notified to the Ministry of Health (MOH) using unique identifying codes. This policy is strongly enforced by law. In addition to notifying the MOH, the Ministry of Interior is also informed about expatriates infected with HIV. HIV-infected expatriates are treated for any acute or life threatening complications and they are then returned back to their homeland. Saudi patients are referred to tertiary care governmental HIV-specialized centers where highly active antiretroviral therapy (HAART) medications and essential laboratory tests, such as HIV viral load and CD4/CD8 counts, are available.

Epidemiological data are collected from patients by the attending physicians on standardized data collection forms. The likely mode of transmission is determined by the attending physician after interviewing the patient and taking complete history regarding high risk behaviors. The mode of transmission is considered to be unknown if the patient fails to admit to any high risk behavior. Collected information is subsequently submitted to the Department of Preventive Medicine in the Central MOH office in Riyadh where all surveillance data are compiled. Annual reports are issued but they are only utilized internally by the concerned officials in the MOH and the Ministry of Interior and they are not made available for the public.

## Results

From 1984 through 2001, a total of 6046 cases with HIV infection were diagnosed; 1285 (21.3%) cases among Saudi citizens and 4761 (78.7%) cases among expatriates. Table 1 shows the demographic data, risk factors, and mortality of the infected patients. The likely mode of HIV transmission among 340 Saudi infected female patients was as follows: blood transfusion, 86 (25.3%) patients; marital sex, 74 (21.8%) patients; maternal transmission to female babies, 27 (7.9%) patients; non-marital sex, 8 (2.4%) patients; intravenous drug abuse, 3 (0.9%) patients; and in the remaining 142 (41.7%) patients the mode of transmission was unknown. Among 1376 non-Saudi HIV infected female patients, the likely mode of transmission was as follows: non-marital sex, 209 (15.2%) patients; blood transfusion, 32 (2.3%) patients; marital sex, 4 (0.3%) patients; intravenous drug abuse, 3 (0.2%) patients; maternal transmission to female babies, 3 (0.2%) patients; and in the remaining 1125 (81.8%) patients the mode of transmission was unknown. A total of 157/1285 (12.2%) Saudi patients had AIDS at the time of HIV diagnosis whereas the rest had either no symptoms (884 patients, or 68.8%) or non-AIDS defining manifestations (244 patients, or 19.0%) such as generalized lymphadenopathy, oral or vaginal thrush, oral hairy leukoplakia, recurrent herpes simplex, herpes zoster, molluscum contagiosum, condyloma acuminata, thrombocytopenia, or aphthus ulcers. Similarly, 600/4761 (12.6%) non-Saudi patients had AIDS at the time of HIV diagnosis, 3618 (76.0%) patients had no symptoms, and 543 (11.4%) had non-AIDS defining manifestations.

Among 4761 HIV-infected expatriates, 3771 (79.2%) patients were from African countries, 624 (13.1%) patients, from Asian countries, 352 (7.4%) patients, from Middle East countries, and 14 (0.3%) patients, from Western countries. Ninety two percent of patients were from 10 countries, namely, Ethiopia (2214 patients or 46.5%), Nigeria (343 patients or 7.2%), Chad (329 patients or 6.9%), Yemen (309 patients or 6.5%), Sudan (267 patients or 5.6%), Eritrea (248 patients or 5.2%), India (219 patients or 4.6%), Somalia (176 patients or 3.7%), Pakistan (152 patients or 3.2%), and Bangladesh (133 patients or 2.8%), and the remaining 371 (7.8%) patients were from 41 other countries.

Table 2 shows the number of HIV cases per 100,000 population by region. HIV cases have been reported from virtually all regions of Saudi Arabia but the highest prevalence was observed in Jeddah and Makkah in the western province (figure 1). Table 3 shows the indications for HIV testing in the Saudi patients. Approximately, one third of cases were identified because of clinical suspicion and the rest of cases were asymptomatic subjects identified following HIV screening for the indications mentioned in table 3.

**Figure 2:**
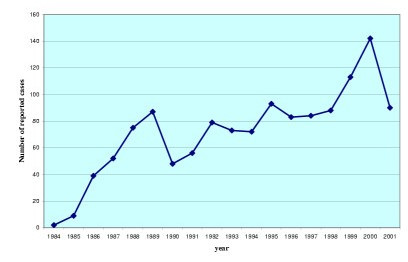
Annually reported HIV infections among Saudi citizens from 1984 through 2001.

**Figure 3:**
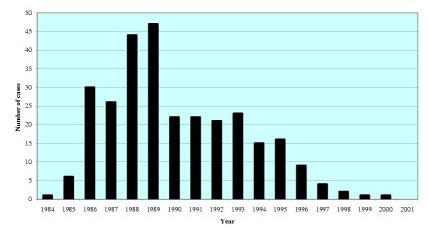
Number of HIV infections transmitted through blood transfusion by year of HIV diagnosis in Saudi Arabia from 1984 through 2001.

Figure 2 shows the annually reported HIV infections among Saudi patients over the surveillance period 1984 through 2001. There has been a gradual increase in the number of cases reported annually but this seems to have plateaued in the period 1997 through 2001. Figure 3 shows the annually reported number of HIV infections transmitted by transfusion of blood or blood products by year of HIV diagnosis from 1984 through 2001. The number of these infections has declined to zero by year 2001. All such infections were due to transfusions administered to patients before 1986.

## Discussion

The prevalence and the annually reported HIV infections in Saudi Arabia were limited. More than three quarters of HIV cases were expatriates. Expatriates are routinely tested for HIV upon arrival to Saudi Arabia and then regularly every 2 years to have their legal residence permits renewed. Therefore, one possible explanation for the higher prevalence of HIV infections among expatriates is that HIV testing rates might be higher among expatriates compared to Saudi citizens. The number of HIV infections diagnosed annually among Saudi citizens gradually increased to reach a plateau of 84 to 142 cases per year in the last 5 years of the study period. The infection was more prevalent in Jeddah than it was in other cities in Saudi Arabia. A possible explanation for this increased prevalence in Jeddah is that this city is the main air and sea port for expatriates who come to work in the western province of Saudi Arabia and for those who come to visit the holy places in Makkah and Madinah. Additionally, the population in Jeddah is multi-cultural and inhomogeneous from the religion point of view.

HIV infection in Saudi Arabia, as the case worldwide, was more common in the age group 20–40 years and predominantly affected men. All modes of transmission were registered but the main mode was heterosexual contact. Even though blood transfusion caused one quarter of HIV cases among Saudi patients, all such infections occurred due to transfusion of blood or blood products before 1986. This risk factor was virtually eliminated since the introduction of routine HIV screening of donated blood and blood products in 1986. The vast majority of Saudi HIV-infected women with identifiable risk factors acquired the infection through blood transfusion or marital sex with their infected husbands. The large proportion of patients with "unknown" risk factors was likely due to deliberate concealment of the actual risk factors, likely to be illegal sex, by the infected patients as non-marital sex and homosexuality are prohibited in Islam, the religion of all Saudi citizens and the vast majority of expatriates living in Saudi Arabia.

The international efforts to control the HIV pandemic have failed to control it on a global scale despite their partial success in many of the developed countries and some of the developing countries. Limitation of resources in underdeveloped countries makes health education and other preventive strategies difficult to implement. Additionally, the limited access to antiretroviral medications in the underdeveloped countries has compounded the problem and perhaps contributed to some extent to the spread of HIV from untreated patients to new victims.

Substance abuse is an increasing problem in Saudi Arabia as it is in the rest of the world [3]. Substances abused include injectable drugs such as heroin and cocaine and non injectable drugs such as cannabis and amphetamine-type stimulants. The estimated annual prevalence of heroin and amphetamine abuse in Saudi Arabia in 2000 as percentage of the population aged 15 and above was 0.01% and 0.002%, respectively [3]. The number of drug abusers annually admitted to detoxification centers in Riyadh, Jeddah, Dammam, and Qassim from 1996 through 2001 ranged from 4740 to 6650 patients with an average annual increment of 5.1% (unpublished data). Several studies were conducted in Saudi Arabia to describe the socio-demographic characteristics, pattern of substance abuse, and prevalence of blood-borne infections among drug abusers. For instance, 799 drug abusers from a voluntary detoxification unit in Jeddah were studied [4]. Sixty eight percent of subjects were under 35 years of age and 64% initiated drugs before age 25. Eighty seven percent used heroin or alcohol and 14% were dependent on more than one drug. Among heroin users, 91% injected the drug. The prevalence of hepatitis C virus infection among these patients was 69% [4]. In another study of 349 drug abusers in Jeddah, 281 (80.5%) subjects were intravenous drug users. The prevalence of HBsAg, anti-HBs, and anti-HBc was 12.6%, 49.0%, and 53.6%, respectively, suggesting that sharing of needles was a common practice [5]. In a more recent study in Jeddah including 1321 drug abusers, 1038 (78.6%) subjects were intravenous drug users and the prevalence of HBsAg and anti hepatitis D virus was 6.1% and 0.8%, respectively [6]. The prevalence of confirmed HIV infection among 2628 drug abusers in Jeddah, of whom 80% were intravenous drug users, was found to be only 0.15% [7]. Among 116 drug users in the Eastern province, 83% of subjects were below 32 years of age, 52.6% were unemployed, and the majority were of intermediate education [8]. Eighty-four percent of the patients abused heroin either alone or in combination with other drugs, 31% used alcohol, 26% used cannabis, and 10% used stimulants. The use of other drugs was rare [8].

Non-marital sex is expected to remain the main risk factor for acquiring HIV infection in Saudi Arabia as it is worldwide for several reasons. The ever-decreasing religious values, the ever-increasing ease of international transportation and communication, and the increasing poverty and unemployment are the main driving forces for non-marital sex, not only in Saudi Arabia but all over the world. Intravenous drug use is also expected to play a more important role for HIV transmission in Saudi Arabia in the years to come as the population of intravenous drug users increases. Preventive strategies should thus be directed towards these risk factors.

Some of the preventive strategies that are advocated and used in other non-Islamic countries such as "Safe Sex" and "Needle Exchange Programs" contradict the Islamic rules and values and as such can not be used as valid and acceptable strategies to prevent the spread of this infection in Islamic countries. The concept of "Safe Sex" basically promotes the use of condoms for non-marital sexual relations, considered in Islamic countries a way of promoting non-marital sex which is absolutely prohibited in Islam. Needle Exchange Programs, likewise, is viewed by Islam as a way of encouraging people engaged in intravenous drug use to continue this prohibited practice.

Strategies to prevent HIV infection in Islamic countries have to abide by the Islamic rules and values. Proposed preventive strategies that can be used in Islamic countries include strengthening of both Islamic and health education, encouraging people to follow and implement the Islamic rules and values that prohibit adultery, homosexuality, and intravenous drug use, and to practice safe sex only through legal marriage. Encouragement of legal marriage is a multidisciplinary task that should involve both governmental and non-governmental charitable organizations and the population at large to cut the cost of marriage and support programs that help the youth to get married. There are several such programs in Saudi Arabia. For example the "Charitable project to help the youth to get married" in Jeddah has helped 2500 young men to get married at a cost of 10 million Saudi Riyals (2.7 million US dollars) collected from donations (personal communication).

Other Islamic aspects that are important in preventing non-marital and irresponsible sexual relationships include the fact that men are allowed to be married to up to four women and the fact that there is no age limit for marriage, thus permitting adolescents to get married. Further, Islam obliges women to cover themselves with the so called "Hijab" or veil and to be segregated from men in educational institutes and other mass-gathering. places. Islam also fights poverty, a driving force for commercial sex and prostitution, through a well established system of obligatory charity, known as "Zakat", and voluntary charity, known as "Sadaqa", taken from the rich people and given to the poor and needy. Additionally, Islam obliges the rulers to eliminate all means and factors that are conducive for indulging in non-marital sex and intravenous drug use such as sex trade, prostitution, and drug smuggling and to implement the Islamic penalties on those involved in such illegal acts. It should be noted, however, that HIV preventive strategies in some Islamic countries do not necessarily abide by the Islamic doctrine and that knowledge, attitude, and practice of Muslims in various Islamic societies do not necessarily conform to Islamic norms.

Unfortunately, there is little published data on the effectiveness of the Islamic preventive strategies in reducing HIV transmission in Muslim communities. According to the United Nations and the World Health Organization data on HIV prevalence in different countries, however, the prevalence of HIV infection in Islamic countries is strikingly low compared to other countries [9,10]. A recent study showed that among 38 sub-Saharan African countries, the percentage of Muslims within countries negatively predicted HIV prevalence [11]. A survey of published journal articles containing data on HIV prevalence and religious affiliation showed that six of seven such studies indicated a negative relationship between HIV prevalence and being Muslim [11].

## Conclusions

Even though the number of HIV cases in Saudi Arabia is still limited, there is a potential for a rapid spread of this virus particularly among the youth. Strategies to prevent the spread of this virus in Saudi Arabia as well as in other Islamic countries should conform to the Islamic rules and values to be successful.

## Competing interests

None declared

## Authors' contributions

TAM designed the study, analyzed the data, and wrote the manuscript. YYM, MHJ, and NSH supervised the data collection and revised the manuscript. All authors read and approved the final manuscript.

## Pre-publication history

The pre-publication history for this paper can be accessed here:



## Figures and Tables

**Table 1:** Demographic data, risk factors, and mortality of HIV infected patients diagnosed in Saudi Arabia from 1984 through 2001.

	Saudi (%)	Non-Saudi (%)	Total
Number of patients	1285 (21.3)	4761 (78.7)	6046
Gender			
Male	945 (73.5)	3385 (71.1)	4330 (71.6)
Female	340 (26.5)	1376 (28.9)	1716 (28.4)
Age groups (year)			
< 5	72 (5.6)	19 (0.4)	91 (1.5)
5 – 14	84 (6.5)	20 (0.4)	104 (1.7)
15 – 19	54 (4.2)	67 (1.4)	121 (2.0)
20 – 29	359 (28.0)	1666 (35.0)	2025 (33.5)
30 – 39	369 (28.7)	2118 (44.5)	2487 (41.1)
40 – 49	188 (14.6)	662 (13.9)	850 (14.1)
50 – 59	86 (6.7)	167 (3.5)	253 (4.2)
≥ 60	73 (5.7)	42 (0.9)	115 (1.9)
Mode of transmission			
Heterosexual contact	487 (37.9)	1352 (28.4)	1839 (30.4)
Blood transfusion	322 (25.0)	186 (3.9)	508 (8.4)
Perinatal	83 (6.5)	19 (0.4)	102 (1.7)
Homosexual contact	32 (2.5)	38 (0.8)	70 (1.2)
Intravenous drug use	17 (1.3)	33 (0.7)	50 (0.8)
Bisexual contact^a^	10 (0.8)	14 (0.3)	24 (0.4)
Unknown	334 (26.0)	3119 (65.5)	3453 (57.1)
Number of patients with AIDS at diagnosis	157 (12.2)	600 (12.6)	757 (12.5)
Mortality	514 (40.0)	Unknown^b^	Not applicable

^a ^Defined as men who have sex with men and women ^b ^Non-Saudi patients were returned back to their homeland.

**Table 2:** Number of HIV cases per 100,000 population by region from 1984 through 2001

Region	No. of Saudi patients	No. of non-Saudi patients	Total	Prevalence per 100,000 population
Jeddah	495	1381	1876	74
Makkah	76	594	670	45
Jizan	42	350	392	39
Dammam	167	474	641	35
Riyadh	217	1075	1292	27
Ahsa	48	139	187	20
Madinah	22	244	266	20
Tayef	66	114	180	19
Baha	45	25	70	18
Joaf	0	31	31	14
Hafr Albaten	4	37	41	14
Hail	5	52	57	12
Najran	11	35	46	12
Abha	44	93	137	10
Qassim	16	72	88	10
Bisha	8	15	23	8
Arar	2	19	21	8
Tabook	17	9	26	4
Qerayyat	0	2	2	2

**Table 3:** Indications for HIV testing in 1285 Saudi HIV-infected patients diagnosed in Saudi Arabia from 1984 through 2001.

Indications	Number	%
Clinical suspicion	438	34
Contacts of patients	190	15
Blood donors	146	11
Hemophilia and other blood disorders	113	9
Before surgery	96	7
Prisoners	84	7
Intravenous drug users	56	4
Patients on hemodialysis	51	4
Pre-employment	46	4
Patients with tuberculosis	36	3
Self-request	19	1
Pregnancy	10	1
Total	1285	100
